# Supplementary material for: Alleviation of Adverse Effects of Drought Stress on Growth and Nitrogen Metabolism in Mungbean (Vigna radiata) by Sulphur and Nitric Oxide Involves Up-Regulation of Antioxidant and Osmolyte Metabolism and Gene Expression
Source: Plants (Basel). 2023 Aug 28;12(17):3082. doi: 10.3390/plants12173082 (PMC10490269; doi:10.3390/plants12173082)
Supplement: Supplementary file 1 [file plants-12-03082-s001.zip › plants-2558997-supplementary.pdf]

Supplement Table S1

Table S1 Sequences for primers used in quantitative real-time RT-PCR.

Gene name	Gene ID	Forward primer (5′-3′)	Reverse primer(5′-3′)	Production size (bp)
Actin	vigna.Vradi03g00210	GCACCACCAGAGAGGAAATAC	TCATACTCAGCCTTCGCAATC	98
Cu-Zn/SOD	vigna.Vradi02g06330	CAACGGTTACTGTTCGCATTACTGGC	GGTCACCCGCATGACGGATTTC	165
POD	vigna.Vradi06g16410	ATGGCAGGTATTGCTACAGTGATGA	CAACCCCTTATGAAGCAATCGTGAA	205
CAT	vigna.Vradi06g16220	CTTCCCCGTCTTTTTCGTCCGT	AAGCCATCCATGTGCCTGTAATCC	198
HSP70	vigna.Vradi11g00650	CTTGTTAACTATCCAAGCTACACCTCTTGC	TTCTTCGGCCTTTTCCCCCTC	187
LEA8	vigna.Vradi08g09340	ATGAGCCAGGAGCAGCCTAAGAGAC	GTCTCTTAGGCTGCTCCTGGCTCAT	126
ruBisCO	vigna.Vradi04g02450	TCCCGGTGTGCTGTTGCAGAGGTA	GGAAGAAGACGATGGGTGAAAGAGTGGTC	122
